# Intervening factors for the initiation of treatment of patients with stomach and colorectal cancer**^1^**

**DOI:** 10.1590/1518-8345.1493.2879

**Published:** 2017-05-15

**Authors:** Thaína Dalla Valle, Ruth Natalia Teresa Turrini, Vanessa de Brito Poveda

**Affiliations:** 2 RN. Student of the specialization course in Intensive Care in Nursing, Centro Universitário São Camilo, São Camilo, SP, Brazil.; 3 PhD, Associate Professor, Escola de Enfermagem, Universidade de São Paulo, São Paulo, SP, Brazil.; 4 PhD, Professor, Escola de Enfermagem, Universidade de São Paulo, São Paulo, SP, Brazil.

**Keywords:** Oncology Nursing, Colorectal Neoplasms, Stomach Neoplasms, Perioperative Nursing, Delivery of Health Care

## Abstract

**Objective:**

to identify the time between symptoms, the request for care and the beginning of treatment in patients with stomach and colorectal cancer as well as the factors that interfere in these processes.

**Method:**

correlational descriptive study, including 101 patients diagnosed with stomach or colorectal cancer, treated in a hospital specialized in oncology.

**Results:**

the 101 patients investigated there was predominance of males, mean age of 61.7 years. The search for medical care occurred within 30 days after the onset of symptoms, in most cases. The mean total time between the onset of symptoms and the beginning of treatment ranged from 15 to 16 months, and the mean time between the search for medical care and the diagnosis was 4.78 months. The family history of cancer (p=0.008) and the implementation of preventive follow-up (p<0.001) were associated with shorter periods between the search for care and the beginning of treatment. Nausea, vomiting, hematochezia, weight loss and pain were associated with faster demand for care.

**Conclusion:**

the longer interval between the search for medical care and the diagnosis was possibly due to the non-association between the presented symptoms and the disease.

## Introduction

Stomach and colorectal cancer stand out among the leading causes of cancer in men and women. An estimated 34,280 new cases of colorectal cancer and 20,520 new cases of stomach cancer happened in Brazil in 2016^1^. Worldwide, colorectal cancer represents the third most common tumor and stomach cancer is the third leading cause of cancer death in both sexes^2^.

Stomach and colorectal cancer are related to several risk factors, both endogenous and exogenous, similar for the two types of tumors: smoking, alcoholism, dietary habits (Low consumption of vegetables, high salt diet, preserved foods and lunch meat), overweight or obesity, physical inactivity, male gender, age older than 50 years, genetic predisposition or family history of cancer. Besides the mentioned factors, in the case of stomach cancer the gastric infection by the bacterium *H. pylori* is pointed out as contributing factor as well as is for colorectal cancer the presence of polyps and history of inflammatory diseases such as ulcerative colitis or Crohn's disease^3^ ^-^ ^4^.

However, most of the cases are diagnosed at an advanced stage, due to the non-specificity of the symptoms in the initial phase of the disease, such as diffuse abdominal pain, asthenia, anorexia and weight loss, compromising healing and reflecting in high morbidity and mortality rates, since the most radical surgery involves the total removal of the stomach^3^.

Regarding colorectal cancer, prevention is anchored in two main aspects: the primary prevention linked to education and lifestyle modification, and the secondary one, consisting in early detection, through the identification of signs and symptoms such as: Hematochezia, change in bowel habit (diarrhea or constipation), weight loss, incomplete intestinal emptying sensation and abdominal pain, together with tests such as fecal occult blood test, sigmoidoscopy and colonoscopy with subsequent biopsy^4^.

Depending on the staging of the disease, the main treatment is surgical. In colorectal cancer, the most radical surgery involves the removal of the large intestine and rectum, leading to the need for colostomy, which social, physical and psychological impact^5^.

Countries such as Japan, England, Canada and the United States of America (USA), which have established programs for the screening of stomach or colorectal cancer, have observed a significant decrease in morbimortality indexes associated with the disease due to the quality of their follow-up methods and the more efficient use of the available diagnostic tests, increasing the capacity of detection and removal of early adenomas and / or carcinomas^6^ ^-^ ^8^.

Diagnosis and staging are key for stomach and colorectal cancer treatment, and the mortality rate and quality of life impairments are directly linked to these two processes. Reaching out in an early stage for a network of health services providing specialized assistance, and the existence of an organized screening program with laboratory studies and procedures for early diagnosis contribute to increasing patients' recovery, avoiding more invasive and extensive procedures^4^ ^-^ ^5^ ^,^ ^9^.

Ideally, the time from diagnosis to tumor surgery should take a maximum of six to eight weeks^10^. However, due to the unequal distribution of healthcare resources and the large geographic extent of Brazil, health care conditions are very diverse, and frequently the demand is much higher than the services capacity, causing delays in diagnosis and treatment compromising the well-being and quality of patient recovery^9^

Despite the existence of international studies that point out the causes and consequences of the delay between the search for medical care, the diagnosis and the beginning of treatment, there is a gap of knowledge regarding this subject in the national literature for colorectal cancer and mainly for cancer of stomach.

Given this epidemiological scenario of high incidence and mortality and the limited number of publications on the subject we aimed to contribute to the identification of weaknesses and strengths of the system, in order to recognize qualities of the service offered to the population and also propose improvement measures. In the hope that this may favor the planning and quality care to patients, we proposed the present study, directed to identify the time between symptoms, the search for care and the beginning of treatment in patients with stomach and colorectal cancer and the factors that interfered in these processes.

## Method

A quantitative descriptive, correlational study performed with 101 patients with diagnosis of stomach or colorectal cancer of both sexes submitted to elective surgery, attended at a hospital specialized in the diagnosis and oncological treatment located in the city of São Paulo - Brazil, from July to December 2014.

Inclusion criteria were: age equal to or older than 18 years and patients diagnosed with stomach or colorectal cancer hospitalized at the surgical clinic of the hospital selected for study to undergo elective surgeries for the treatment of cancer. Patients who had difficulties in understanding or communicating with the researcher and patients submitted to intestinal transit reconstruction surgery after the end of treatment were excluded, as these patients were distant from the difficulties of the start of the treatment, hindering the recall of the situations experienced until there.

For the sample calculation, at the time of conducting this study, the only investigation similar to this (evaluation of the time of onset of symptoms until diagnosis), had been conducted among patients with lung cancer^10^. Thus, despite the differences between tumor types, it was decided to use them, since it was considered that the system of public health care used by patients was the same, added to the fact that the cited research contained the necessary statistical data, allowing us to adequately estimate the number of observations to be made.

Thus, the sample calculation was based on the national study findings^10^ in which the mean time between onset of symptoms and the first visit was 110 days with a standard deviation of 72.5 days. Thus, considering the time between symptoms and the first visit, it was estimated that it would be necessary to observe 90 patients, in order to obtain the average time of search for health care, with a margin of error of 15 days and a confidence interval of 95%.

The project met the conditions of resolution 466/2012^11^, being approved by the Research Ethics Committee, under protocol number 24731414.3.0000.5392.

Data were collected through a data collection instrument, which contained sociodemographic data and aspects related to diagnosis and treatment.

Three oncology nurses, who analyzed the data collection instrument to evaluate its ability to achieve the proposed objectives and their clarity, suggested changes that were accepted by the authors.

The data collection itself occurred through an interview of the patient or person in the preoperative period, after which they received explanations about the research objectives and expressed their agreement through the signing of an Informed Consent Term. The patients' records were also used as a source of complementary information, such as staging and clinical data.

The analysis of the data was made in a descriptive and inferential ways, through the *software Statistical Package for the Social Science (*SPSS), version 20.0. The results were presented using frequency distributions and statistical descriptive measures such as arithmetic mean, standard deviation (SD), median, minimum and maximum values for the quantitative variables. The dichotomous variables were evaluated using the exact test of *Fisher* while the continuous variables were analyzed using *Mann Whitney*. The delimited level of significance was α equal to or less than 0,05.

## Results

A total of 101 patients investigated, a mean age of 61.7 years (SD=12.94 years), predominantly male (53.5%) (Table 1) and residing in the city of São Paulo (43.6%).

**Table 1:** Sociodemographic data of patients with stomach or colorectal cancer undergoing elective surgery. São Paulo, SP, Brazil, 2014

Variable		n	%
Sex			
	Male	54	53.5
	Female	47	46.5
Color			
	Caucasian	55	54.5
	Mixed	30	29.7
	Black	16	15.8
Marital Status			
	Married	53	52.5
	Widower	17	16.8
	Single	15	14.9
	Divorced	13	12.9
	Lives with a partner	3	3.0
Ocupation			
	Retired	47	46.5
	Self-employed	19	18.8
	Employed	15	14.9
	Pensioner	11	10.9
	Unemployed	7	6.9
	Others	2	2.0
Education			
	1 to 4 years of study	44	43.6
	5 a 8 years of study	25	24.8
	Never attended school	13	12.9
	9 to 11 years of study	12	11.9
	12 years or more of study	7	6.9
Home			
	Home owner	76	75.2
	Renter	17	16.8
	Other	8	7.9
Total		101	100

The patient was responsible for the family income in 69 (68.3%) of cases, in 16 (15.8%) of the situations the spouse was cited, and, in sequence, children(10; 9.9%) or other people (6; 6%). The referred income was from two to five minimum wages (47; 46.5%), followed by a minimum wage (35; 34.7%), in four (4%) income was higher than six salaries and 15 (14.9%) did not know how to report or report income less than a minimum wage. Regarding nutritional status, 12 (11.9%) presented Body Mass Index (BMI) portraying low weight, 43 (42.6%) normal weight, 33 (32.7%) overweight and 13 (12.9%) were obese. Nine (8,9%) interviewees reported being alcoholic, while 46 (45.5%) never used alcohol and 46 (45.5%) discontinued use, 16 reported being smokers (15.8%) and 41 (40.6%) ex-smokers. Comorbidities were present in 51 (50.5%) subjects, highlighting Systemic Arterial Hypertension (SAH) (21; 20.8%), heart disease (18; 17.8%) and the combination between SAH and Diabetes Mellitus (DM) (15; 14.9%) (Tabela 2).

**Table 2:** Clinical-surgical variables of patients with stomach or colorectal cancer submitted to elective surgery. São Paulo, SP, Brazil, 2014

Variable		N	%
Chronic Disease			
	Systemic Arterial Hypertension	21	20.8
	Heart disease	18	17.8
	Systemic Arterial Hypertension and Diabetes Mellitus	15	14.9
	Diabetes Mellitus	02	1.4
	Other comorbidities	19	18.8
Diagnosis			
	Rectum Cancer	56	55.4
	Stomach Cancer	30	29.7
	Colon Cancer	15	14.9
Surgical indication			
	Abdominal reosigmoidectomy	47	46.5
	Partial / total gastrectomy	23	22.7
	Exploratory laparotomy for drainage and / or biopsy	10	9.9
	Partial / total colectomy	7	6.9
	Abdomino-perineal amputation of rectum in oncology	4	3.9
	Excision of anus-rectal cancer / lesion	4	3.9
	Peritonectomy	3	2.9
	Others	3	2.9
Staging			
	IA	18	17.8
	IIA	17	16.8
	IIB	16	15.8
	IIIA	12	11.9
	IV	5	5.0
	IIIC	4	4.0
	IIIB	3	3.0
	Cancer *in situ*	3	3.0
	IB	2	2.0
	Not recorded in medical records	21	20.8

Diagnoses of rectal cancer as well as rectosigmoidectomy surgeries were the most frequent. Staging of the tumors, by means of the classification system TNM ("T": information on the primary tumor; "N": Lymph node involvement, and; "M": Existence of metastases), revealed that the highest incidence were IA (18; 17.8%), IIA (17; 16.8%) and IIB (16; 15.8%). Worth of note is the presence of five (5%) patients on stage IV, which indicates presence of metastases (Table 2).

Only 19 (18.8%) interviewed had preventative exams (Endoscopy, colonoscopy, fecal occult blood and tomography), of these, 13 (68,4%) already had some diagnosis of previous cancer; four (21%) performed it by medical indication; one (5,3%) due to the family history of cancer in these sites and (5,3%) preferred to carry it out "voluntarily".

Concerning family history of cancer, 62 (61,4%) of patients had a family history of cancer, 37 (36,6%) denied cases and two (2%) could not answer as they did not know their biological family. In all, 100 family members with a diagnosis of cancer were mentioned, being 22 (22%) of stomach, 19 (19%) colorectal and 59 (59%) in other locations. Ten interviewees had at least two family members with stomach or colorectal cancer.

Due to the accomplishment of the preventive screening examinations, seven (6,9%) patients had no symptoms before the diagnosis of cancer.

Symptoms were associated by the respondents in 35 (34.7%) cases to some disease, other than cancer; 18 (17.8%) patients associated the symptoms with eating habits and 13 (12.9%) did not relate the symptoms to anything. Only nine (8.9%) linked the symptoms to cancer. The main symptoms reported can be seen in Table 3.

**Table 3:** Distribution of the symptoms identified by the interviewees. São Paulo, SP, Brazil, 2014

Symptoms reported	N	%
Epigastric and / or abdominal pain	47	46.5
Hematochezia	47	46,5
Weight loss	46	45.5
Appetite loss	19	18.8
Diarrhea	19	18.8
Constipation	19	18.8
Fatigue	17	16.8
Anal bleeding	17	16.8
Vomit	13	12.9
Feeling of incomplete bowel emptying	11	10.9
Nausea	12	11.9
Colic	17	16.8
Gastric fullness	10	9.9
Flatulence	10	9.9
Others	15	15.0

The search for medical care occurred up to 30 days after the onset of symptoms in 67 (66.3%) cases, and in 34 (33.7%) it took more than 30 days to find a health center. Of these, 24 (70.6%) needed more than 90 days to seek for help, six (17.6%) took from 30 to 60 days and four (11.8%) took 60 to 90 days.

Rgarding the reason for the longer delay in the search, 25 (73.5%) answered that they did not associate the symptoms with a health problem, three(3%) had never attended a health service before, two (2%) referred fear, two (2%) did not know how to respond and (1%) for not being able to leave work.

The number of referrals between the primary care location sought by the patient and the institution that performed the oncological treatment itself was evaluated. Thus, the mean number of referrals to treatment was 1.14 (SD=0.98), with a minimum of zero and a maximum of three. From the first place in which they sought medical attention until the attendance at the specialized institution, 33 (32.7%) they needed a referral; 32 (31.7%) were referred directly to the institution; 26 (27.7%) had two referrals and 10 (9.9%) three referrals or more.

Thus, on average, the time elapsed between the onset of symptoms and the search for medical care was 2.15 months; 4.78 between the search for medical care and the diagnosis; 4.06 months between diagnosis and initiation of treatment and the total time between symptoms and onset of treatment was, on average, 15.16 months (Table 4). Some patients reported not remembering the periods they waited between stages (7.9%).

**Table 4:** Time between onset of symptoms, search for treatment, diagnosis and initiation of treatment. São Paulo, SP, Brazil, 2014.

Intervals	n	Time (months)		
		Mean(±SD)	Medium	Min-Max
Between symptoms and seeking medical care	93	2.15 (±3.94)	1	0-25
Between seeking medical care and diagnosis	100	4.78 (±7.19)	2	0-43
Between diagnosis and initiation of treatment	101	4.06 (±4.05)	3	0-34
Between symptoms and initiation of treatment	93	15.16(±10.97)	12	0-67

Age was related to the time between diagnosis and initiation of treatment (QT, RT or surgery) (p=0.044). That is, the older the age, the longer the time for seeking medical care. Systemic arterial hypertension had a significant association with the reduction of the time between the search and the medical diagnosis (p=0.012). Family history of cancer was significantly associated with the shortest time between onset of symptoms and treatment seeking (p=0.08). Preventive follow-up presented significant associations between the shortest time elapsed in relation to the search for treatment and the diagnosis (p=0.07) and also between the symptoms and the beginning of treatment (p<0.001).

Significant associations were observed between the variables: weight loss and the shortest time elapsed between the onset of symptoms and the search for medical care (p=0.010); Nausea and the time between onset of symptoms and initiation of treatment (p=0.024); Vomiting and reducing the time elapsed between the onset of symptoms and the search for medical care (p=0.042) and initiation of treatment (p=0.005); Hematochezia and the shortest time between the beginning of treatment and surgery (p=0.006) and pain at the shortest time between diagnosis and initiation of treatment (p=0.003).

There was a significant association between the patients who started the search for treatment at the Basic Health Unit (UBS) and the longer time elapsed between the search and the diagnosis (p=0.009). The income (p=0.689), education (p=0.394), sex (p=0.564), marital status (p=0.842) religion (p=0.552) were not associated with the search for help after the onset of symptoms and did not interfere in the time intervals studied.

## Discussion

The majority of the patients were male; with a mean age of 61.7 years; mainly smokers or ex-smokers, and, alcoholics or ex-alcoholics; married; with income between two and five minimum wages; had up to four years of study, the most frequent diagnosis being rectal cancer. It should be noted that the older the person, the longer the time between the onset of symptoms and the search for treatment.

The results concur with the scientific literature regarding the mean age of the patients and the predominance of this type of cancer among males^2^ ^,^ ^12^ ^-^ ^13^, alcohol and tobacco users^14^ ^-^ ^16^ and with low educational level, as aspects associated with delays longer than 30 days until the first treatment, demonstrating that not only clinical aspects, but also cultural factors interfere in the treatment search process^12^.

In the present investigation, most of the patients analyzed sought medical care up to 30 days after the onset of symptoms, however, the time between the search for medical care and the beginning of treatment occurred on average in 8.84 months. In this sense, in an effort to accelerate access to health services, several countries around the world have sought to implement specific screening programs for stomach and colorectal cancer.

In the 1990s, the Danish government began to implement a program that recommended that the time between suspected and performed exams in patients investigated for colorectal cancer should occur in a maximum of 14 days, plus a further 14 days between the diagnosis and treatment, thus totaling a period of 28 days between suspicion and treatment^17^. In the United Kingdom, as of 2000, the "two-week referral" recommendation was introduced for patients with suspected colorectal cancer, if they met predefined criteria for age, signs and symptoms^18^.

Recent research analyzing the implementation of the British program showed that the number of diagnoses of colorectal cancer showed a significant increase, compared to periods before its beginning, however, no differences were found regarding the stage of the diagnosed tumors or the survival period in two years^18^.

This aspect differs from the implementation of screening programs for gastric cancer, especially in Asia, which has contributed to the diagnosis of the disease in the early stages and therefore increased the survival of these patients, thus generating better results than those obtained in the West, in relation to the same disease^19^.

Only recently, Brazil, through Law no. 12,732^20^, enacted in 2012, recommended that cancer treatment, regardless of cancer type, should be started within 60 days after diagnosis. It is important to emphasize that the term recommended by Brazilian law takes into account only the period between admission and treatment of the patient in the institution specialized in oncology, therefore, does not evaluate the necessary referrals until this service.

The experiences of the patients investigated, the period between the diagnosis or suspected diagnosis and effective treatment took an average of four months. It is important to highlight that, in the current study, the entire process of search for treatment reported by the patient, that is, from the first symptoms to the oncological treatment itself, is described in the specialized institution.

It is noteworthy that, although screening programs are capable of increasing the diagnostic capacity of oncological disease^18^ ^-^ ^19^, different from that occurring in gastric tumors^19^, the scientific literature does not seem to be able to demonstrate the capacity of these initiatives to impact on the mortality associated with colorectal cancer^18^ ^,^ ^21^ ^-^ ^22^.

In this sense, there is evidence that independent of guarantees of equal access to the health system and diagnosis, through *screening* for the disease, most diagnoses are associated with the presence of signs and symptoms, especially rectal bleeding, abdominal pain, or change in bowel habits and fatigue, which seems to culminate in more advanced stages of colorectal neoplasia^21^ ^-^ ^22^.

Recent literature review has indicated controversies in the analysis of the role of delayed diagnosis, colorectal cancer survival and disease stage^23^. There seems to be consensus in stating that signs and symptoms considered worrisome by patients, such as bleeding and abdominal pain, lead to a more agile search for health care, generating faster diagnoses, as in cases of rectal tumors. Diseases with less specific symptoms, such as those occurring among patients with colon cancer, require a longer investigation period until their definitive diagnosis, culminating in more advanced stages of the disease^23^ ^-^ ^24^.

In this investigation, it was also observed that the time between presenting the first symptoms and seeking medical care was over two months and the mean time between symptoms and the beginning of treatment was approximately 15 months. In addition, patients who took more than 30 days to seek care in 73.5% of the cases did not associate their symptoms with any health problem, highlighting, according to the literature, the importance associated with the patient's attitudinal aspects in the attribution of value to the signs and symptoms felt, motivating their agility in the search for the health service^12^ ^-^ ^24^.

Despite the limitations of access to information regarding the stage, it was observed in this investigation that a minority of cases had the *in situ* disease. The diagnosis of cancer *in situ* is difficult because of the large number of asymptomatic patients or with atypical symptoms in gastric and colorectal cancer, even in countries with good screening*,* such as Japan and the United States of America (EUA)^6^ ^,^ ^13^ ^,^ ^25^.

However, it is worth mentioning that after the implantation of the screening program for these neoplasias in the USA, the rate of Americans over 50 who have already undergone colonoscopy has tripled in the last 11 years, contributing to the fall of 3.9% of the disease in this age group, even so, only 40% of patients with colorectal cancer were diagnosed with the disease *in situ* in 2014^13^. Regarding patients with gastric cancer, the five-year survival for the disease worldwide is 20%, while in Japan, for diseases diagnosed between stages I and II, the survival rate reaches 70%^25^.

In addition, it is interesting to note that another aspect that may have influenced the search for the health service may be linked to the frequency in men of stomach and colorectal cancer^2^ ^,^ ^12^ ^-^ ^13^ as it is well known that women tend to adhere better to health programs, e.g. the breast cancer screening program.

It was also observed that aspects that collaborated in the agility of care were observed, such as the significant association between arterial hypertension and the shorter time to diagnosis, suggesting that patients with chronic diseases and those who follow up at a health service have greater agility in the detection of new diseases, as well as those who are being tracked for prevention due to family or personal history of cancer, which certainly contributes to a greater chance of better prognosis. Thus, monitoring for chronic diseases favors greater access to the service and health professionals, thus increasing the chances of patients having their symptoms recognized, leading to the suspicion of the disease and referral for exams^10^.

Recommendations on the need for a control program for gastric and colorectal cancer in Brazil have existed since the 1990s, however, the difficulties associated with the high cost of detection tests and their invasive nature can not be ignored, together with the need for training of health professionals beginning in their professional education, mainly those who work in primary care, in order to identify signs and symptoms of suspicion^9^.

Thus is evidenced the importance of launching a screening program, the dissemination of information on stomach and colorectal cancer, as well as the integration among health services, the decrease in waiting time by examinations and the investment in professional training in the health area for the more agile detection of cases and better quality of life of the patient.

The limitations of the study lie in the geographical delimitation of the data, when investigating the patients referred to an important cancer treatment center in a single municipality.

Another aspect to be pointed out is the lack of national studies that investigate the subject, especially among gastrointestinal tumors, which can also be presented as a limitation, since it weakens the comparisons of the results found to the national reality.

Thus, the need to carry out similar investigations in other regions of the country is reinforced, producing studies that will allow the identification of barriers and solutions, contributing to the elaboration of a well-structured Brazilian public policy for the treatment of potential cases of stomach cancer and colorectal cancer.

## Conclusion

The major cause of delay in the search for medical care was the non-association between the symptoms presented as cause of illness. Family history of cancer and preventive follow-ups were significantly related to shorter periods of search and initiation of treatment. The period between symptoms manifestation and getting treatment lasted on average 15.16 months, and this period may be justified due to the need in the majority of the sample, for at least one or two referrals to the place of effective treatment.
